# Transcriptomic profiling of cotton *Gossypium hirsutum* challenged with low-temperature gradients stress

**DOI:** 10.1038/s41597-019-0210-7

**Published:** 2019-10-09

**Authors:** Zhi-Bo Li, Xiao-Yan Zeng, Jian-Wei Xu, Rui-Hai Zhao, Yi-Nong Wei

**Affiliations:** 1 College of Agronomy, Shihezi University, Shihezi, 830032 China; 2 Key Laboratory of Oasis Eco-agriculture of Xinjiang Production and Construction Corps, Shihezi, 832003 China

**Keywords:** RNA sequencing, Abiotic, Transcriptomics

## Abstract

*Gossypium hirsutum*, a cotton species widely cultivated around the world, is a typical cold-sensitive crop. Low-temperature (LT) stress is one of the main environmental stressors that can affect growth and the quality of cotton fibers. LT is also a major challenge for cotton survival, growth maturity and geographical distribution. However, few genome-wide transcriptional response and profiling datasets are available to explore the LT-tolerant mechanism of cotton. This study treated *G*. *hirsutum* with four LT gradients (control at 25 °C and cold temperatures at 4 °C, 10 °C and 15 °C) for 24 hour to generate 12 RNA-Seq datasets (three biological replicates per treatment) with approximately 280 million clean reads per dataset. The quality of the datasets obtained in the current study was validated through a series of quality checks including verification of RNA sample quality and RNA-Seq read quality. Data analyses included novel gene discovery, global gene expression profiling and quantitative real-time PCR. This is the first study to report genome-wide transcriptomic datasets for cotton in response to LT exposure.

## Abstract (summary)

**Table:** 

Measurement(s)	transcription profiling assay
Technology Type(s)	RNA sequencing
Factor Type(s)	treatment temperature • biological replicate
Sample Characteristic - Organism	Gossypium hirsutum

Machine-accessible metadata file describing the reported data: 10.6084/m9.figshare.9878594

## Background & Summary

Low-temperature (LT) is a major challenge for the growth and survival of plants generally and can have significant impact on the productivity of economically important crops^1^. LT below a critical threshold can damage many plants, resulting in multiple complex symptoms such as water loss, abnormal growth or browning in leaves, wilting or deformity in flowers as well as shed and shriveled fruit^2^. Recently, the gene expression response in plants to LT has been extensively described in the study of plant adversity-tolerance biology. This includes numerous studies investigating genome-wide transcriptomic levels in response to LT treatment using RNA sequencing (RNA-Seq) technology. For example, Jiao *et al*. (2016) performed transcriptomic analysis of peach (*Prunus persica*) stigma under LT stress using digital gene expression profiling^2^. The authors identified 276 and 966 differentially expressed genes (DEGs) in peach stigma treated with LT (−2 °C) for 4 h and 26 h, respectively, when compared to the control maintained at room temperature. These DEGs were primarily related to processes such as response to temperature stimulus, carbohydrate metabolism, cytoskeletal maintenance, hydrolase activity and primary metabolic pathways^2^. Wang *et al*. (2018) used RNA-Seq technology to analyse the genome-wide transcriptomic profile of chrysanthemum (*Dendranthema grandiflorum*) in response to LT stress^3^. A total of 7,583 DEGs were identified from 36,462 annotated unigenes and these DEGs were significantly enriched to gene ontology (GO) and Kyoto Encyclopedia of Genes and Genomes (KEGG) pathways involving low temperature sensing and signal transduction, membrane lipid stability as well as reactive oxygen species (ROS) scavenging and osmoregulation^3^. However, dynamic changes in gene expression of cotton under LT stress remain largely unknown.

Generally, exposure to cold temperatures caused changes in gene expression, followed by increased levels of hundreds of proteins involved in protective roles against damage. For example, one of these important pathways is related to the C-repeat-binding factor and dehydration responsive element-binding factor 1 (CBF/DREB1) regulation^2,4^. The genes encoding CBF/DREB1 proteins belong to the APETALA2/ethylene-responsive (AP2/ERF) transcription factor superfamily. AP2/ERF transcription factors bind to the C-repeat/dehydration-responsive (CRT/DRE) elements present in the promoters of a large number of cold-regulated (COR) genes that have been widely thought to promote plant tolerance in response to abiotic challenges^2,5^. In addition to the COR genes regulated by AP2/ERF transcription factors, the abundance of dehydrins also significantly affected cold tolerance of plant. The dehydrin GhDhn1 was involved in LT and drought tolerance in cotton^6^. Considering the complexity of the genetics, physiology and biology for LT tolerance, the comprehensive list of genes and pathways involved in LT response remain to be identified and analysed, which can provide a valuable resource for understanding of molecular mechanisms regarding LT tolerance in plants.

Cotton *G*. *hirsutum* belongs to the family Malvaceae, *Gossypium* genus, is native to the subtropical zone, and enjoys warmth and sunshine^7^. The optimum temperature for growth is 20–30 °C^7^. With an average temperature of lower than 15 °C during the day, the growth of cotton is adversely affected. China’s cotton is mainly planted in the North-Western inland region (cotton area), the Yellow River basin and the Yangtze River basin cotton area^7^. In recent years, increased frequency of LT and cold damage during the cotton growth period has gradually increased the impact of LT chilling damage on cotton production^7^. Researchers are paying more attention to various physiological changes of cotton under LT stress.

LT stress induced dramatic transcriptomic changes in the cotton. For example, Shan *et al*. (2007) found that the up-regulated expression of genes encoding for transcription factors and C-repeat binding factors/dehydration-responsive element binding proteins (GhDREB1) increased cold tolerance and improved plant growth and development through negative regulation of gibberellic acid^8^. Similarly, cold stress treatment (10 °C or less) induced expression of the phospholipase D-α (PLDα) gene, normally suppressed under control temperatures (25 °C or 22 °C), following acclimatisation at 17 °C before applying the cold treatment. Differential expression levels of transcript isoforms of this gene were recorded under cold acclimation and cold stress temperatures^9^. However, these studies have focused on the expression levels of a single gene or a small selection of genes. Little is known about transcriptional response to cold stress at the genome-wide level. This lack of transcriptomic data for cotton under LT stress hinders our comprehensive understanding of the molecular responses in cotton when exposed to cold and what changes may afford cold tolerance.

**Fig. 1:**
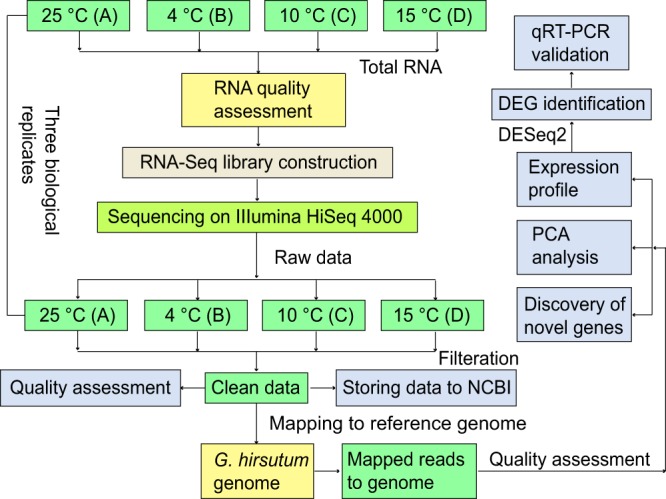
Overview of the experimental design and analysis pipeline. The raw data were assessed and filtered using the FastQC and Trimmomatic tool, respectively. The clean reads were mapped to the *G*. *hirsutum* reference genome using HISAT2. DEGs were identified using DESeq2.

No. 36 Zhongmian Institute cotton, *Gossypium hirsutum*, is an LT-tolerant breed that is widely planted in Xinjiang Province, the most important inland cotton producing area in China^10^. Previous investigations on the physiological response of cotton seedlings under low temperature stress showed that most of the enzymes involved with LT tolerance of *G*. *hirsutum*, such as peroxidase, superoxide dismutase, cinnamol dehydrogenase and ascorbic acid peroxidase, *etc*.^11^. Therefore, No. 36 Zhongmian Institute breed and 24 h are the optimal experimental material and treatment time for LT studies of *G*. *hirsutum*. Here, No. 36 Zhongmian Institute *Gossypium hirsutum* was subjected to a gradient of LT treatments (4, 10 and 15 °C) as well as the control at 25 °C, by simulating their natural environment under controlled conditions. Sampling time points were set for 24 hour after various LT treatment and three biological replicates were produced for each temperature treatment. A group of 12 RNA-Seq datasets containing approximately 280 million sequence reads were generated using the HiSeq. 4000 platform (Illumina, USA). RNA-Seq read quality and the global gene profiles were assessed (Fig. 1) to ensure the reliability of our datasets. These transcriptomic data provides a useful resource for further research that will contribute to a comprehensive understanding of the molecular mechanisms for LT tolerance in *G*. *hirsutum*.

## Methods

### Plant materials and experimental design

*G*. *hirsutum* seeds with full grain were selected for this study. Concentrated sulfuric acid was used to delint the seeds for 3 minutes followed by washing with water for 2–3 times. The seeds were placed evenly in a Petri dish with double layers of filter papers (80 seeds per dish) and the right amount of moisture was ensured. The dishes were placed in a light incubator (Sanyo, Japan) under 16/8 h of light/dark illumination, 28/26 °C for day/night, relative humidity of 70% and cultured for 36 h. At this time, when the cotton radicles were about 1 cm in length, the germinated seeds were moved to the nursery boxes for seedling. 1,000 g of washed sand that was dried at 120 °C, was put into each nursery box. Next, 200 mL of tap water was added into the nursery boxes to soak the sand. Germinated seeds with the same growth were selected and transplanted into the nursery boxes (28 seeds per box). Then, 400 g of sand was sprinkled evenly into each nursery box to bury the seeds. When three true leaves grew, the seedlings with the same growth were selected for control or cold temperatures at 25 °C (Group A, control), 4 °C (B), 10 °C (C), and 15 °C (D) for 24 h. Fifteen seedlings were collected for each treatment temperature (4, 10, 15, and 25 °C) in per biological replicate. The experiment was independently repeated three times to create three biological replicates. All samples were immediately frozen in liquid nitrogen after 24 hours of temperature exposure and stored at −80 °C.

### RNA extraction and Illumina sequencing

Total RNA was extracted from the leaves of each seedling using the RNA prep Pure Plant Kit (No. DP441; Tiangen Co. Ltd, China) and an RNase-free DNase Set (Qiagen, Germany) was used to digest residual genomic DNA. Each of fifteen plants was independently used for RNA extraction, and then the total purified RNA was equally pooled as one biological replicate for each temperature treatment. Prior to pooling, RNA concentration and quality of each sample was determined using a BioPhotometer Plus spectrophotometer (Eppendorf, Germany) and gel electrophoresis. Only RNA samples with an absorbance ratio at 260/280 nm between 1.8 to 2.0 and structural integrity verified by gel electrophoresis was used for further experiments. Library construction was prepared using the TruSeq RNA Sample Prep Kit (Illumina, CA, USA). Briefly, ribosomal RNAs (rRNAs) were removed from the total RNA and the remaining RNAs were fragmented into short fragments of approximately 200–500 nucleotides (nt). First-strand complementary DNA (cDNA) was synthesised from fragments using random hexamer primers with dUTP, which was substituted for dTTP during the synthesis of the second-strand. A single adenine was added to the ends of the short fragments after purification and subsequently connected with adapters. Finally, the second-strand was degraded using uracil-N-glycosylase (UNG). The cDNA library was sequenced at the HiSeq 4000 platform (Illumina, USA) at the Breeding Biotechnologies (http://www.biobreeding.com.cn/, China) using standard procedures to generate paired-end reads of 150 bp.

### Data filtering and assessment

The quality assessment of raw data was performed using FastQC v0.11.6^12^. Next, the raw sequence reads were trimmed using Trimmomatic (v0.32) (http://www.usadellab.org/cms/?page=trimmomatic), where sequencing adapters, low-quality bases were excluded. The exclusion criteria were (1) adaptor sequences of raw reads, (2) bases with quality score below 20, (3) reads containing over 10% “N” and (4) reads with read length shorter than 75 nt. Additionally, Deconseq (v0.4.3, http://deconseq.sourceforge.net/) was used to remove potential contaminated reads from sources like rRNA and virus. Te reference of sources sequences was downloaded from FTP server of NCBI (fp://fp.ncbi.nih.gov/). The statistics summary of clean reads is presented in Table 1. Furthermore, the quality of the clean data was summarized using MultiQC v1.325^13^. Clean reads of all the samples were submitted to the NCBI SRA database^14^.

**Table 1:** Statistics of *Gossypium hirsutum* transcriptomes in this study.

Sample	Description	Clean reads	Clean read rate (%)	All mapping rate (%)	GC (%)	Q20 (%)	Biosample accession
A1	25 °C, control_1	22,692,301	99.14	85.77	43.83	96.57	SAMN10339005
A2	25 °C, control_2	22,563,909	98.97	86.04	43.71	95.23	SAMN10345423
A3	25 °C, control_3	24,312,847	99.26	85.92	43.67	95.46	SAMN10359076
B1	4 °C LTTS_1	23,877,422	99.44	86.52	44.07	96.42	SAMN10319703
B2	4 °C LTTS_2	21,473,129	99.17	86.27	43.85	95.34	SAMN10334135
B3	4 °C LTTS_3	20,238,400	99.28	85.99	44.34	96.42	SAMN10348835
C1	10 °C LTTS_1	23,519,223	99.51	87.12	43.72	96.02	SAMN10362740
C2	10 °C LTTS_2	24,120,983	99.22	86.84	43.77	96.49	SAMN10335321
C3	10 °C LTTS_3	21,499,287	99.08	86.95	43.99	96.34	SAMN10357421
D1	15 °C LTTS_1	24,416,803	98.74	87.21	43.46	96.15	SAMN10373579
D2	15 °C LTTS_2	24,698,886	98.92	87.07	43.71	95.89	SAMN10373829
D3	15 °C LTTS_3	22,903,376	99.13	87.34	43.84	96.15	SAMN10352489

Clean data rate = Clean read number/Raw read number * 100%. Q20 (%) indicates the percentage of bases with quality scores more than or equal to 20. LTTS: low-temperature treated samples for 24 hour.

### Gene quantification and assessment of transcriptomic library quality

The clean reads were mapped to the *G*. *hirsutum* genome (ftp://ftp.bioinfo.wsu.edu/species/Gossypium_hirsutum/, BGI version) using HISAT2^15^ with default parameters. The RNA-seq by Expectation Maximization (RSEM) tool was used to calculate the fragments per kilobase of genes per million fragments (FPKM) based on the quantity of mapped reads for each unigene^16^. Sample reproducibility was assessed among the three biological replicates based on the FPKM values of each unigene using Pearson’s correlation coefficient (*r* values). Differentially expressed genes (DEGs) were screened using DESeq2 software, which is an R package used for identification of DEGs in general for transcriptomes containing biological replicates^17,18^. Genes were considered as DEGs if the fold changes (FC) were ≥2 (|log2 ratio| ≥1) and the *p*-values were less than 0.01 (Wald test in DESeq2), corrected by the Benjamini-Hochberg method (false discovery rate, FDR). Randomness test of mRNA fragmentation, where higher randomness of transcript fragmentation indicated a more uniform coverage of reads on transcript and higher-quality of samples, was performed. In addition, RNA-Seq read coverage saturability over the gene based on the mapping results was assessed to test whether sequencing data was adequately representative.

### Discovery of novel genes

Reads mapped to *G*. *hirsutum* reference genome were assembled using Cufflinks programme^19^ and subsequent assembled genes were searched against the reference genome using Blastn tool. The assembled genes (excluding sequences encoding for peptides with less than 50 amino acid residues or contain only a single exon) that did not match anything in the reference genome were annotated as novel genes. These annotated novel genes improved genomic annotation of the *G*. *hirsutum* reference genome.

### Analysis of quantitative real-time PCR

Extracted total RNA of samples were also used for quantitative real-time polymerase chain reaction (qRT-PCR) validation. First-strand cDNA was synthesized using the RevertAid™ First Strand cDNA Synthesis Kit (Thermo Scientific, USA) according to manufacturer’s instructions. Primer Express software (Applied Biosystems, USA) was used to design gene-specific primers for qRT-PCR (see the file “qRT-PCR primers used in this study” on Figshare^20^). The cDNA (100 ng/μl) were quantified using SYBR® Premix Ex Taq II Kit (Takara, Japan) on the ABI PRISM 7500 Real-Time PCR System (Applied Biosystems, USA). Fifteen DEGs were selected randomly for the qRT-PCR validation, normalized against the house-keeping gene GAPDH^21^. Each biological replicate was measured in triplicate technically and relative gene expression levels were calculated using the 2^−ΔΔCT^ method^22,23^. Pearson’s correlation coefficient (*r*) between RNA-Seq and qRT-PCR results were calculated using IBM SPSS 22 software.

## Data Records

The project (Bioproject ID: PRJNA498759) was deposited into the *NCBI Sequence Read Archive* database^14^. Combined Supplementary Information can be found on Figshare^20^.

## Technical Validation

### RNA quality control

The quality of the extracted total RNA played a key role in the construction of RNA-Seq libraries and downstream transcriptomic analyses. In particular, high RNA integrity (RIN) values ensured that adequately unique RNA-Seq reads were possible^24^, while lower RIN values could lead to potential bias in gene expression analyses. All the RNA samples used for RNA-Seq library construction had RIN values of more than 7.0. This ensured that high quality reads were generated by RNA-Seq for further analyses, as was commonly used in similar studies^23^. The quality summary of each of the RNA samples is shown in Table 2.

**Table 2:** RNA sample quality for each sample.

Sample	RIN	28S/18S	OD260/280	OD260/230
A1	7.4	1.8	1.9	2.3
A2	7.5	1.7	1.9	2.2
A3	7.2	1.8	2.0	2.5
B1	7.4	2.0	1.9	2.1
B2	7.6	2.3	1.8	2.4
B3	7.4	1.9	1.7	2.7
C1	7.4	2.1	2.1	2.4
C2	7.5	1.8	1.9	2.8
C3	7.6	1.9	2.0	2.2
D1	7.2	1.8	2.0	2.7
D2	7.4	2.1	2.1	2.2
D3	7.5	1.9	2.0	2.6

### Quality validation

**Fig. 2:**
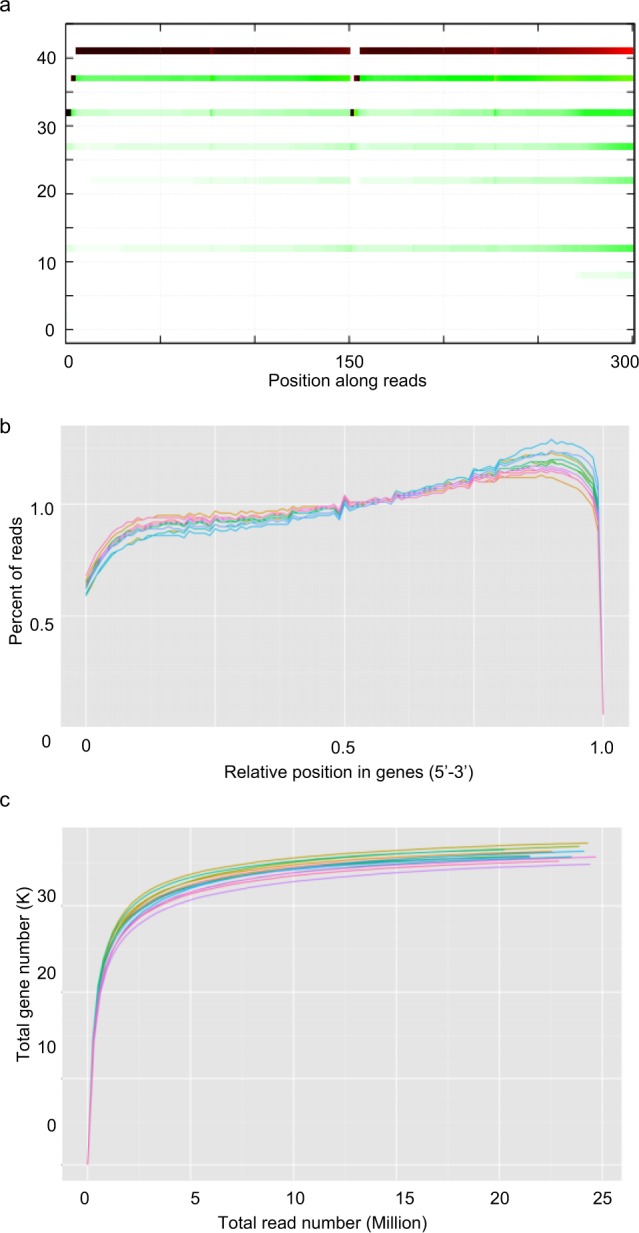
Quality assessment metrics for RNA-Seq data. (**a**) Per base sequence quality. (**b**) Distribution of mapping reads on the reference genes. (**c**) Saturation simulation of RNA-Seq data.

After filtering the raw reads, a high rate of clean reads from each sample was achieved, ranging from 98.74% to 99.51%, and the mapped rate was above beyoned 85% for each library (Table 1), implying successful library construction and RNA sequencing. These clean reads have been submitted to the Sequence Read Archive (SRA) database of NCBI (the Bioproject accession numbers PRJNA498759). Assessment of the read base quality by FastQC is presented in Fig. 2a. The majority of per base quality scores was above 30, with many above 40, indicating high-quality RNA-Seq data. The GC content of all samples was stable with the distribution ranging from 43.46–44.34%. Read-mapping qualities of the 12 samples including mapping rates, the randomness of transcript fragmentation and read coverage saturability were also checked. The mapping rates of all the samples to the reference genome were above 85% (Table 1). The distribution of reads on the reference genome showed smooth curves of fragmentation randomness (Fig. 2b), indicating that the genome was sequenced uniformly and there were no degradation of RNA samples. The results of read coverage saturability are illustrated in Fig. 2c. All the curves extended to the right with shallow slopes that gradually plateaued, indicating that increased sequencing led to saturation and there were fewer novel genes detected. These analyses demonstrated that the sequencing data in this study was adequately representative and valid.

Pearson’s correlation coefficient (*r*) (see the file “Pearson’s correlation coefficient values among samples” on Figshare^20^) and principle component analysis (PCA) (see the file “PCA” on Figshare^20^) of the data profiles from all 12 samples revealed high correlation among all the samples. Data from samples within the same LT treatment condition showed a higher correlation than the data between treatment groups. The results indicate an effective LT treatment process, high-quality bioinformatics analyses, and minimisation of sample variation as a result of individual bias. A total of 4,406 novel genes were discovered in this study and their respective GFF files and sequences are published with this article (see the file “New gene sequences” on Figshare^20^).

**Fig. 3:**
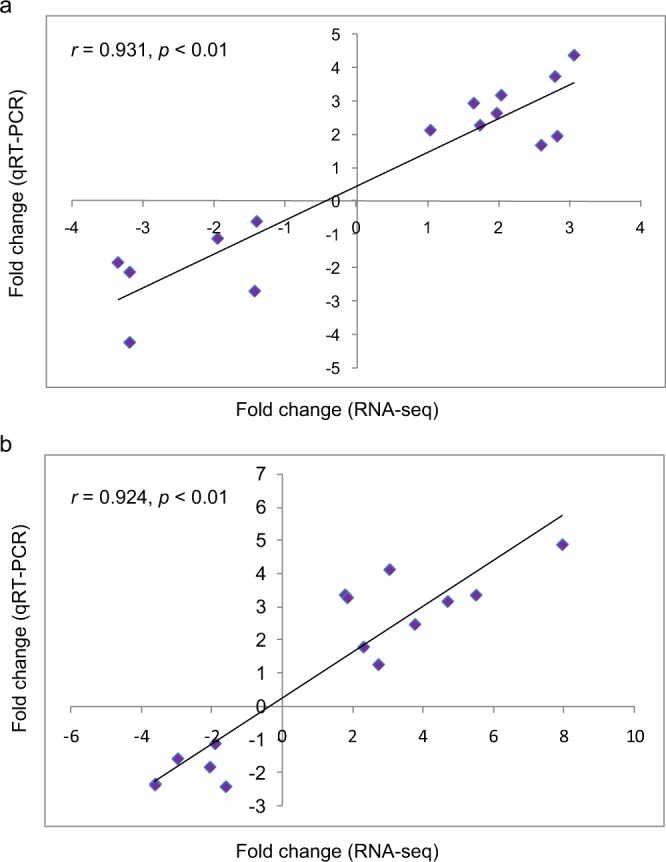
Correlation between relative fold changes in expression based on RNA-Seq and qRT-PCR analyses. Each dot indicated the relative expression fold-change between the control and treatment groups by RNA-Seq (x-axis) and qRT-PCR (y-axis). Three technical replicates were performed for each biological replicate. (**a**) Control vs. *G*. *hirsutum* treated with 4 °C LT and (**b**) Control vs. *G*. *hirsutum* treated with 10 °C LT.

In addition, a total of 2,477 (A vs. B), 3,188 (A vs. C) and 2,950 (A vs. D) DEGs were identified for the various LT treatments (see the file “Differentially expressed genes” on Figshare^20^). The results of the qRT-PCR analysis of 15 randomly selected DEGs showed high correlations of fold-change in expression level between the RNA sequencing and qRT-PCR results for A vs. B (*r* = 0.931, *p* < 0.01) and A vs. C (*r* = 0.924, *p* < 0.01), confirming the high accuracy of the RNA sequencing and analyses in this experiment (Fig. 3a,b).
